# Implementing antibiotic stewardship in high-prescribing English general practices: a mixed-methods study

**DOI:** 10.3399/BJGP.2022.0298

**Published:** 2023-02-21

**Authors:** Sarah Tonkin-Crine, Monsey McLeod, Aleksandra J Borek, Anne Campbell, Philip Anyanwu, Céire Costelloe, Michael Moore, Benedict Hayhoe, Koen B Pouwels, Laurence SJ Roope, Liz Morrell, Susan Hopkins, Christopher C Butler, Ann Sarah Walker

**Affiliations:** Nuffield Department of Primary Care Health Sciences, University of Oxford, Oxford; National Institute for Health Research (NIHR) Health Protection Research Unit in Healthcare Associated Infections and Antimicrobial Resistance, University of Oxford, Oxford.; NIHR Health Protection Research Unit in Healthcare Associated Infections and Antimicrobial Resistance, Imperial College London, London; Centre for Medication Safety and Service Quality, Pharmacy Department, Imperial College Healthcare NHS Trust, London; and NIHR Imperial Patient Safety Translational Research Centre, Imperial College London, London.; Nuffield Department of Primary Care Health Sciences, University of Oxford, Oxford.; NIHR Health Protection Research Unit in Healthcare Associated Infections and Antimicrobial Resistance, Imperial College London, London.; Centre for Medical Education, School of Medicine, Cardiff University, Cardiff.; Institute of Cancer Research, London.; Primary Care Population Sciences and Medical Education, Faculty of Medicine, University of Southampton, Southampton.; Primary Care and Public Health, Imperial College London, London.; Nuffield Department of Population Health, University of Oxford, Oxford.; Nuffield Department of Population Health, University of Oxford, Oxford.; Nuffield Department of Population Health, University of Oxford, Oxford.; UK Health Security Agency, London.; Nuffield Department of Primary Care Health Sciences, University of Oxford, Oxford.; Nuffield Department of Medicine, University of Oxford, Oxford; NIHR Health Protection Research Unit in Healthcare Associated Infections and Antimicrobial Resistance, University of Oxford, Oxford; and NIHR Oxford Biomedical Research Centre, Oxford.

**Keywords:** antibiotic prescribing, antimicrobial resistance, antimicrobial stewardship, behaviour change, communication, C-reactive protein, delayed prescription, implementation, point-of-care testing

## Abstract

**Background:**

Trials have identified antimicrobial stewardship (AMS) strategies that effectively reduce antibiotic use in primary care. However, many are not commonly used in England. The authors co-developed an implementation intervention to improve use of three AMS strategies: enhanced communication strategies, delayed prescriptions, and point-of-care C-reactive protein tests (POC-CRPTs).

**Aim:**

To investigate the use of the intervention in high-prescribing practices and its effect on antibiotic prescribing.

**Design and setting:**

Nine high-prescribing practices had access to the intervention for 12 months from November 2019. This was primarily delivered remotely via a website with practices required to identify an ‘antibiotic champion’.

**Method:**

Routinely collected prescribing data were compared between the intervention and the control practices. Intervention use was assessed through monitoring. Surveys and interviews were conducted with professionals to capture experiences of using the intervention.

**Results:**

There was no evidence that the intervention affected prescribing. Engagement with intervention materials differed substantially between practices and depended on individual champions’ preconceptions of strategies and the opportunity to conduct implementation tasks. Champions in five practices initiated changes to encourage use of at least one AMS strategy, mostly POC-CRPTs; one practice chose all three. POC-CRPTs was used more when allocated to one person.

**Conclusion:**

Clinicians need detailed information on exactly how to adopt AMS strategies. Remote, one-sided provision of AMS strategies is unlikely to change prescribing; initial clinician engagement and understanding needs to be monitored to avoid misunderstanding and suboptimal use.

## INTRODUCTION

Antibiotic prescribing reduced by 7.5% in England between 2015 and 2019, but significant regional variation in antibiotic use continued despite adjusting for case mix.^1^^–^^3^ In England in 2019, 71% of antibiotics were prescribed in general practice.^1^ This includes a substantial contribution to total broad-spectrum antibiotic use^1^ that is widely acknowledged to be associated with development of antimicrobial resistance.

Effective antimicrobial stewardship (AMS) interventions exist. Interventions that have successfully reduced antibiotic prescribing in trials include education-based strategies for GPs, point-of-care (POC) tests, for example, POC C-reactive protein (CRP) tests (POC-CRPTs), use of delayed prescriptions, training in enhanced communication skills for clinicians, audit and feedback, and clinician reminders.^4^^–^^8^ POC tests provide additional clinical information to a prescriber to support them to make a diagnosis and treatment decision. A CRP result can indicate whether a patient is likely to benefit from antibiotics or not. Reviews indicate that interventions that are multifaceted (targeting more than one behaviour change mechanism), multilevel (targeting more than one stakeholder group), and multicondition (targeting several types of infection) are more likely to be effective than interventions with a single focus.^4^^,^^9^^,^^10^ Qualitative work has identified that GPs want interventions that decrease diagnostic uncertainty, provide patient-centred care, and are easy to implement.^11^

**Table:** How this fits in

An intervention to support the implementation of three evidence-based antimicrobial stewardship strategies was evaluated in nine high antibiotic prescribing general practices in England. General practice teams received intervention materials and chose to use them in substantially different ways in real-life settings, outside of trial conditions. Antimicrobial stewardship strategies are complex interventions that require sufficient understanding and engagement by clinicians for successful adoption and use, to obtain the full benefit in reducing antibiotic prescribing. This study highlights that remote, one-sided delivery of AMS strategies should be done cautiously to avoid misunderstanding and suboptimal use.

Despite this, effective interventions have not been routinely implemented, with often only temporary improvements in prescribing rates even in trial sites.^12^^,^^13^ Although existing interventions target individual factors that directly influence behaviour they may fail to fully account for organisational factors that can influence intervention implementation.^14^

Many AMS strategies have been available in English general practice (for example, TARGET toolkit, Antibiotic Guardian) and there have been improvements in antibiotic prescribing; however, some practices are still prescribing relatively high quantities of antibiotics, which may not be fully explained by their patient population.^1^^,^^2^ These practices may benefit from more support in implementing AMS strategies. Rather than specify one strategy, giving practices a choice of approaches may help teams find what works for them. Furthermore, providing interventions that are complementary in their mechanisms of action may also provide benefit, with two or more strategies being better than either alone.^7^

There is limited research on uptake and effect of AMS strategies in English general practice outside of trials. Often this research has taken the form of quality improvement initiatives in a single practice. The authors of the current study previously described the co-development of an implementation intervention, with primary care staff and citizens, for general practice to help improve use of three AMS strategies that previous trials have shown to be effective and safe.^15^ This study aimed to investigate the use of the implementation intervention in high-prescribing practices and its effect on antibiotic prescribing.

## METHOD

### Implementation intervention

The authors of the current study co-developed an implementation intervention^15^ to support the use of three AMS strategies:

- enhanced communication skills with or without a patient leaflet;
- delayed prescriptions; and
- POC-CRPTs.

**Figure 1.**
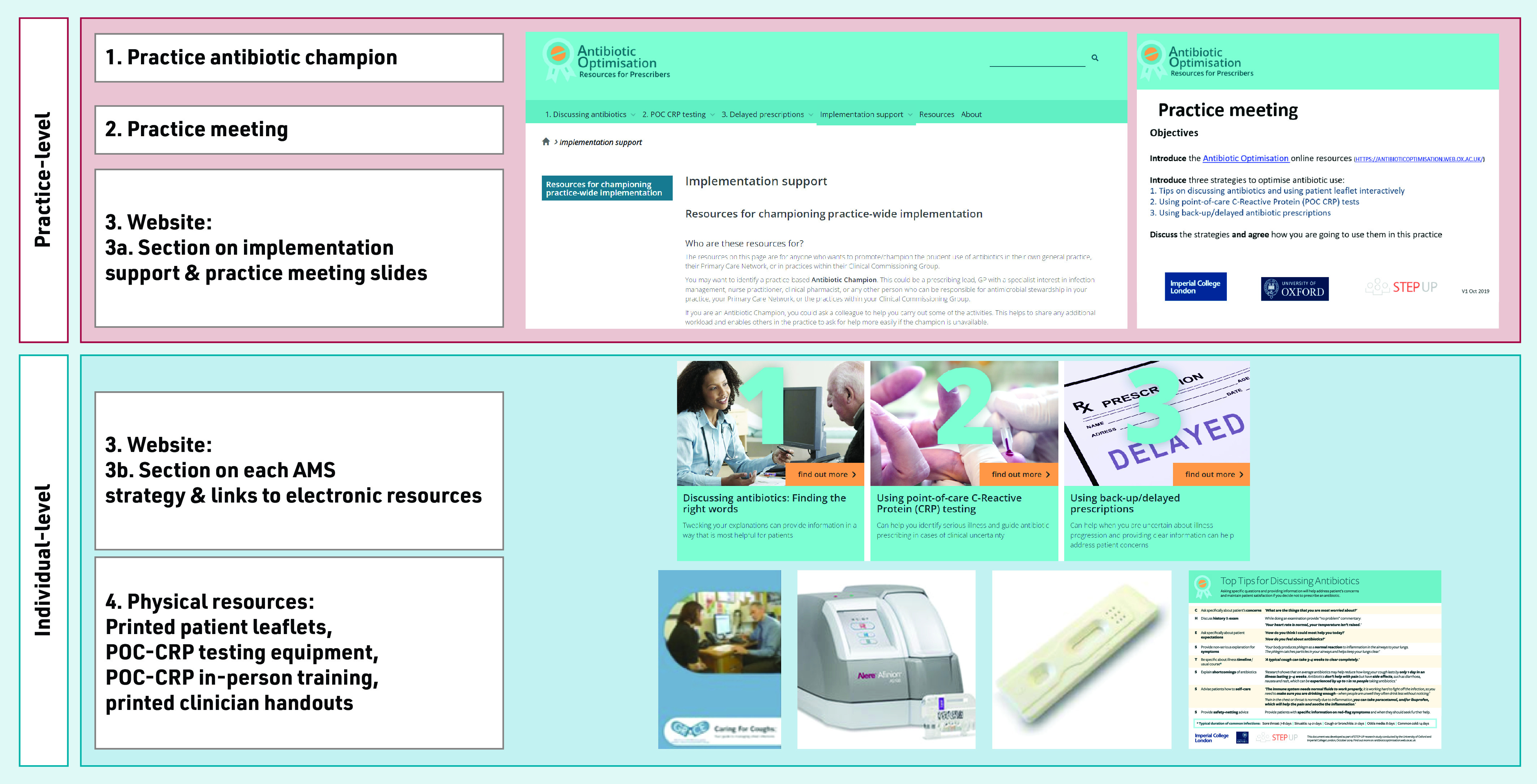
*Components of the antibiotic optimisation implementation intervention.AMS = antimicrobial stewardship. POC-CRP = point-of-care C-reactive protein.*

The intervention was designed to be brief, provided choice in uptake of strategies, and be delivered remotely with minimal input from the research team. The implementation intervention included (Figure 1):

- identifying a champion;
- holding a practice meeting to agree a practice-wide approach to implementation;
- an ‘antibiotic optimisation’ website including: implementation support for the champion and sections on three AMS strategies for clinicians; and
- physical resources: patient leaflets, POC-CRPT equipment, clinician handouts.

Each practice was offered an Afinion^TM^-2 analyser, 60 Afinion CRP cartridges, and 30 SureScreen CRP lateral flow tests. Practices had access to in-person training on use of the Afinion-2 analyser. Printed copies of patient leaflets and clinician handouts were provided.^15^ Practice teams were advised to use the intervention materials, as they wished, and encouraged to have follow-up practice meetings. Teams were able to select which AMS strategies they wished to implement.

### Setting and participants

The aim was to recruit 8–10 practices from the 20% of highest antibiotic prescribing practices in England (based on antibiotic items per Specific Therapeutic group Age-sex Related Prescribing Unit [STAR-PU] from ePACT data in 2018)^16^ and in areas local to the research team. Practices were contacted by email or post. Practices that expressed interest were selected to ensure variation in location (region and urban/rural), number and type of healthcare professionals (HCPs), and local area deprivation (based on the overall English Index of Multiple Deprivation 2015 by postcode).^17^

Practices were offered £1000 after study set-up and another £1000 at the end of the study when at least 70% of eligible HCPs had completed surveys at each timepoint.

### Data collection and analysis

#### Practice-level antibiotic prescribing

The primary outcome was total antibiotic prescriptions per practice, as reported in the NHS Business Service Authority dataset of all prescribing centres in England, summarised over time (count/month).^18^ Forty-five practices from the same clinical commissioning groups as intervention practices were selected as a control. Practices were matched on pre-intervention trends in overall antibiotic prescribing rate, practice list size, and prevalence of comorbidities (asthma, cancer, chronic kidney disease, cardiovascular disease, and diabetes). A difference-in-difference analysis was used to estimate intervention effects, comparing change in the differences in observed outcomes between intervention and control groups, across pre-intervention and post-intervention periods.

#### Use of intervention materials

Website use was monitored through Google Analytics. The website address was known only to the practice teams. Practices could request additional CRP cartridges/tests or printed materials. Orders from each practice were recorded.

#### Surveys

Surveys were sent at three timepoints: baseline, 2 months, and 12 months. Surveys asked about views on antibiotic prescribing, the three strategies, and satisfaction with the intervention materials (Supplementary Information S1). The Consolidated Framework for Implementation Research and the Normalisation Measure Development questionnaire were used to guide question development.^18^^,^^19^ HCPs consented at the start of each survey. Associations between responses at baseline and follow-up surveys were assessed using χ^2^ tests.

#### Interviews

The plan was to interview two HCPs from each practice at 6 and 12 months, to make it feasible for practice teams to participate in the qualitative interviews. The person liaising with the study team in each practice identified participants. Interviews explored views of AMS strategies, intervention materials, and antibiotic prescribing (Supplementary Information S2). Interviewees gave verbal consent before each interview. Interviews were audio-recorded and transcribed verbatim; field notes were also made. The first and the third author used deductive framework analysis and developed an a priori framework based on the topics of interest.^20^ Transcripts were coded, using NVivo software (version 12), to assign data to pre-existing categories, informed also by field notes. Data that did not fit these categories were given their own categories and the framework developed.

## RESULTS

Ninety-seven practices were invited and 15 expressed interest (15% response rate). Nine practices participated (Table 1). The study ran from November 2019 for 12 months. The COVID-19 pandemic from March 2020 onwards had an impact on UK general practice and study activities paused after this date. Results are focused on the period up to March 2020.

**Table 1.** Summary characteristics of the nine general practices participating in the study

**Characteristic**	**Value**
**Location, practices, *n***	
West Midlands:	
Birmingham	2
Warwick	1
Worcester	1
Thames Valley and South Midlands:	
Milton Keynes	1
South Oxfordshire	1
Wycombe	2
Aylesbury vale	1

**Deprivation,^a^ practices,*n***	
High deprivation	4
Low deprivation	5

**Urban/rural practices,*n***	
Major conurbation	2
City and town	3
Town and fringe	3
Village	1

**Antibiotic prescribing across four quarters in 2018 based on total antibiotic items per STAR-PU,^b^ range (median)**	0.27–0.35 (0.30)

**Number of GPs per practice, range (mean)**	3–11 (5)

**Patient list size, range (median)**	2439–13 995 (5317)

a *Based on Index of Multiple Deprivation 2015 (range 1–10 with 1–2 high deprivation and 7–10 low deprivation).*

b *Specific Therapeutic group Age-sex related Prescribing Unit (STAR-PU) from electronic Prescribing Analysis and Cost Tool (ePACT) data, 2018. For all practices in England; median 0.23 (range 0.0003–2.27).*

### Antibiotic prescription data

**Figure 2.**
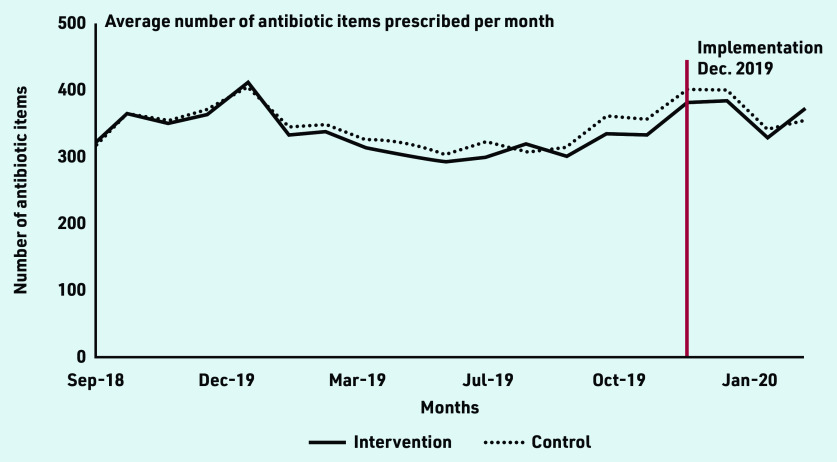
*Number of antibiotic items prescribed in the intervention and control practices before and after implementation.*

Data from between September 2018 and March 2020 were analysed for nine intervention and 45 control practices. It was assumed implementation occurred in December 2019, giving practices 4 weeks from the start of the study to adopt the AMS strategies they chose. The mean number of antibiotic items per month for the intervention group was 331 (SD 174) and 367 (SD 182) pre-and post-implementation, respectively, and 340 (SD 172) and 374 (SD 189), respectively, in control practices (Figure 2).

A time series plot of the total number of antibiotic items prescribed by each intervention practice over the study period indicated that in five practices prescribing increased after the intervention period and in four prescribing stayed relatively the same (Supplementary Figure S1). There was no evidence of differences in covariate distributions between intervention and control groups, or pre- and post-implementation (Supplementary Table S1). In the difference-in-difference regression, there was no evidence of an effect of the implementation intervention on total antibiotic prescribing (Supplementary Table S2), or prescribing of individual antibiotics (Supplementary Table S3).

### Survey and interview participants

Practices identified 81 HCPs to complete surveys (Table 2). The baseline survey was completed October to November 2019, 2-month follow-up between December and January 2019–2020. The 12-month survey is not reported here as it was conducted after March 2020.

**Table 2.** Overview of responses to surveys and interviews by practice

**Practice**	**Eligible practice HCPs,^a^ *n***	**Baseline survey responses October 2019, *n* (of those, prescribers, *n*)**	**2-month follow-up survey responses December 2019, *n* (of those, prescribers, *n*)**	**Interviews February/March 2020, *n* (job title)**	**Interviews October/November 2020, *n* (job title)**
**A**	4	3 (2)	2 (1)	1 (nurse)	1 (GP)
**B**	10	8 (3)	10 (3)	1 (GP)	0
**C**	5	5 (5)	3 (3)	2 (GP)	1 (GP)
**D**	9	7 (1)	8 (2)	0	1 (GP)
**E**	13	13 (11)	12 (10)	0	1 (GP)^b^
**F**	14	11 (5)	7 (3)	2 (ambulatory clinician and GP)	1 (GP)
**G**	7	6 (3)	5 (3)	1 (GP)	1 (GP)
**H**	11	5 (5)	5 (5)	0	1 (GP)^b^
**J**	8	8 (4)	9 (5)	2 (GP)^b^	1 (GP)
**Total**	81	67^c^ (39)	62^c^ (35)	9	8

a *The number of eligible HCPs in practices changed over the duration of the study. Numbers shown were as reported by practice contacts at baseline.*

b *One interview with a clinician who was not the antibiotic champion.*

c *One responder did not identify which practice they were from. HCPs = healthcare professionals.*

Thirteen HCPs participated in interviews: nine in February–March 2020 and eight in October–November 2020 (Table 2). Nine participants completed one interview; four completed interviews at both timepoints. Interviews lasted 18–39 min (mean 28 min).

### Prescribers’ views on antibiotic prescribing

Survey data indicated that views on antibiotic prescribing changed little between baseline and 2-month follow-up (Supplementary Table S4). However, when asked about their prescribing at 2 months, most prescribers (28/35, 80%) believed their antibiotic prescribing had improved since the start of the study.

### Engagement with implementation and the three AMS strategies

All practices confirmed they had identified an antibiotic champion, completed their practice meeting, attended POC-CRPT training, and had received intervention materials. Eight practices accepted Afinion and SureScreen POC-CRP equipment. Practice J opted out of using Afinion POC-CRP equipment as they did not consider it feasible for clinicians to share one machine.

Table 3 shows an overview of implementation engagement based on interviews. It was found that survey responses sometimes contrasted with interview data (discussed below), and interview data were prioritised; these gave more detail as to how a practice engaged.

**Table 3.** Summary of engagement with the implementation intervention materials and AMS strategies by practice

**Practice**	**Study briefing phone call completed? (by whom)**	**Usual role of antibiotic champion (nominated/volunteered)**	**Practice meeting completed? (focus of meeting)**	**Champion engaged with website?**	**AMS strategies chosen**	**Additional resources ordered**
**A**	Yes (champion)	Principal GP and nurse (both volunteered)	Partially (POC-CRPT)	No	POC-CRPT	Afinion CRP cartridges
**B**	No	Salaried GP (nominated)	Partially (study set-up not AMS strategies	No	POC-CRPT	None
**C**	Yes (champion)	Salaried GP (nominated)	Partially (POC-CRPT)	No	POC-CRPT Leaflets	Leaflets
**D**	Yes (practice manager)	Partner GP (nominated)	Partially (unknown)	No	POC-CRPT Leaflets	Leaflets
**E**	Yes (champion)	Advanced nurse practitioner (volunteered)	Yes (3 AMS strategies and audit)	No	POC-CRPT Leaflets	Afinion CRP cartridges Leaflets
**F**	Yes (practice manager)	Partner GP and ambulatory clinician (both volunteered)	Partially (POC-CRPT, leaflets)	No	POC-CRPT Leaflets	Afinion CRP cartridges Leaflets
**G**	Yes (champion)	Principal GP (volunteered)	Yes (3 AMS strategies)	No	POC-CRPT Leaflets	Afinion CRP cartridges Leaflets
**H**	No	Partner GP (nominated)	Partially (unknown)	No	POC-CRPT	None
**J**	Yes (champion)	Partner GP (volunteered)	Yes (3 AMS strategies and used presentation slides)	Yes	POC-CRPT Leaflets Delayed prescription Communication strategies	SureScreen tests Leaflets

a *From interaction with study team. Green: optimal engagement with implementation activity/resource. Orange: partial engagement with implementation activity/resource. Red: no engagement with implementation activity/resource. AMS = antimicrobial stewardship; CRP = C-reactive protein. POC-CRPT = point-of-care CRP test.*

Champions were asked to have familiarised themselves with all materials, including the implementation section of the website, to have held a practice meeting discussing the three AMS strategies, and to have chosen which strategies to use. Practices differed in how champions did these activities.

### Champions and practice meetings

There were 11 champions across nine practices (Table 3). Seven champions answered the survey at 2 months. Six agreed that they were able to engage and encourage their colleagues to use the intervention resources; one was neutral. Champions were satisfied with how each AMS strategy was being implemented in their practice: communication strategies with leaflets (5/7); POC-CRPTs (7/7); and delayed prescriptions (7/7). Of the remaining 55 survey responders, 42/55 (76%) knew who their champion was; of those who did not, five were from practice H. Of those who knew their champion, over half agreed their champion encouraged colleagues to engage with intervention materials (29/55, 53%).

Interviews highlighted variation between practices in champion engagement with intervention materials. Five practices had clinicians who volunteered for the champion role. They were enthusiastic, often senior clinicians, with allocated time to dedicate to the role:

*‘*[She] *has one session a week to do administrative work and she had the enthusiasm to do it, and she took it on, but it was on the proviso that she wasn’t having to do all the work, she passed over information to us but it was as long as we were all in on it. ’*

(J2, GP, senior partner [the letter after each quote refers to the practice and the number, the participant]).

Four champions had been nominated for the role and appeared less engaged, with less time to give:

*‘*[The antibiotic champion] *role came along with several other roles that were coming in, like children’s safeguarding, women’s health lead, opioid prescribing lead and we’re not a practice that has a lot of doctors. So, there’s a lot of roles that needed filling and there’s only so few hands, so somebody had to take something and it just fell to me. ’*

(B1, GP)

Champions emphasised that they needed sufficient time to undertake the role of engaging others successfully, which was only possible for some individuals:

*‘*[You need] *to select someone who willingly signs up, that shows motivation and if you can get the practice to commit to giving them some admin time every week, that way there’s structured time for them to engage. But if the* [champion] *is bogged down by admin and extra work, the motivation may be there but the energy isn’t and I think that may have been the same in my case. ’*

(B1, GP)

*‘I’m making sure that the* [resources] *are available to all the doctors. I’ve spoken to them and answered questions. Just trying to keep it in people’s minds really so they’re aware of it and they are thinking about the project and using the resources we’ve got … because it’s so busy here, it’s very easy for things to slip back into old routines …* [the champion role] *just means that the responsibility’s on one person to keep it current, else it will just get put in the back of people’s minds.’*

(F1, non-prescriber)

Champion engagement influenced the content of practice meetings. Three champions (practices E, G, J) reported discussing all three AMS strategies with their teams. Other champions had not engaged with the website and only focused on physical materials (POC-CRPTs and leaflets, Table 3):

Interviewer: *‘Can you tell me more what happened in that meeting? ’*

Participant: *‘*[Doctor] *did the front of house bit and I did the training bit. But all the team, both clinical and non-clinical were briefed on* [the CRP machine]. ’

Interviewer: *‘Did you focus on the CRP testing mainly in that meeting? Or were there other things? ’*

Participant: *‘It was a CRP meeting, also obviously about antibiotics — for looking at how they’re used generally within practice. I think *[CRP] *gives the doctors some evidence supporting their decisions really. ’* (F1, non-prescriber)

One champion (Practice J) focused on all three strategies and used the meeting slides provided on the website. The team decided how they would use delayed prescriptions and distributed patient leaflets and SureScreen tests to each consultation room:

*‘Everyone who’s responsible for prescribing was at the meeting, we were all in agreement that we have to all be prescribing with the same ideals so that we could improve things.’*

(J2, GP)

#### Website

Data showed that the website had 75 new users. Of all survey responders, 52% (32/62) had not visited the website at 2 months, 24% (15/62) had visited it once, and 21% (13/62) had visited it twice or more. Of 30 responders who had visited the website, most found the content helpful: communication strategies (19/30, 63%), delayed prescription (20/30, 67%), and POC-CRPTs (23/30, 77%).

In interviews, only one champion (Practice J) reported spending time on the website. Other interviewees were either not aware of the website or had only briefly looked at it, reporting that they already felt familiar with the content. Instead, champions had focused on the physical materials that had been posted to practices.

Some interviewees thought that the website was aimed at patients rather than themselves. Despite this, participants generally thought a website was an appropriate format for them to access information easily. Time was felt to be the main barrier to use:

‘[The website] *will be a very useful thing, because all of us, even in our daily practice we use, I possibly use a dozen websites a day. So a website is good for quick referencing, so that you’re always one or two clicks away from things, so I would say it’s a very good idea. It’s a lot better than printed out information or email information.’*

(B1, GP)

Interviewer: *‘What, in your view, are the barriers to people going and looking at those resources online? ’*

Participant: *‘Time, to be honest, time. If you come in on a Monday morning and you’ve got four hundred prescriptions to do, and that’s, even before the day is out you’ll have got another two or three hundred. And to get through them is so fast and takes such a long period of time. I think the biggest obstacle in anything we have to achieve in primary care is time. ’* (H2, GP)

#### Communication strategies and patient leaflets

At the 2-month survey, most prescribers (28/35, 80%) responding to the survey were confident that they could effectively communicate a ‘no antibiotic’ decision without affecting patient satisfaction; this was up from baseline (19/39, 49%) (Supplementary Table S4). Prescribers reported that using patient leaflets interactively in consultations (29/35, 83%) had helped reduce antibiotic prescribing. At baseline 20/39 (51%) prescribers reported using patient leaflets in respiratory tract infection (RTI) consultations; at 2 months this had increased to 27/35 (77%) prescribers.

When commenting on discussing antibiotics with patients, interviewees referred to the need to ‘educate patients’. Interviewees from practices J and F had engaged with the communication strategies and discussed specific techniques that they found useful:

*‘I’ve learned* [about] *using the resources to educate patients and explain to them that something lasting for four to seven days can be quite normal.’*

(J2, GP)

*‘The* [handout] *that gives points on how you can talk about antibiotics in a different way. I’ve been really advocating that, amongst the practice.’*

(F2, GP)

Other prescribers felt they needed additional strategies (for example, POC-CRPTs) to back up explanations about no antibiotic decisions.

Interviewees were enthusiastic about leaflets and liked the evidence-based options provided, saying that they supported discussion although highlighted that leaflets were only used if close to hand:

*‘If it’s not to hand it doesn’t really happen. A bit out of sight, out of mind, maybe we need to change that.’*

(D1, GP)

*‘We have them on the desk. We did that with all the clinical rooms. They are literally just in front of you. ’*

(J1, GP)

#### Delayed prescriptions

At baseline most prescribers were confident that they could explain a delayed prescription to a patient and this did not change at 2 months (Supplementary Table S4). At 2 months, most prescribers reported using delayed prescriptions (29/35, 83%).

Most prescribers (25/31, 81%) agreed that increased use of delayed prescriptions had helped to reduce antibiotic use in their practice (of the six prescribers who did not use delayed prescriptions, two still answered the question about the effect of delayed prescriptions in their practice). Prescribers used various formats: gave to patient with advice to delay (19/29, 66%), post-dated prescription (12/29, 41%), asked to collect from agreed location (8/29, 28%), and contact practice again (6/29, 21%).

In contrast to the survey responses, interviewees reported that they did not think delayed prescriptions were useful and did not use them frequently or at all. Clinicians felt patients would take antibiotics immediately regardless of what they were told, and discussed delayed prescription formats as ways of preventing access:

*‘I didn’t* [use delayed prescriptions] *very much because I suspect if I gave my patients prescriptions they’d go off and take them straight away. ’*

(E1, GP)

*‘I got the dispensary to show me how to do the delayed scripts by changing the date … by changing the script to the twenty-third, they can’t get it before the twenty-third.’*

(C1, GP)

Three practices were dispensing practices; in one of these they did not use delayed prescriptions at all for this reason:

*‘We’re a dispensing practice, patients pick medication up on their way out. I genuinely think we all feel it doesn’t work.’*

(D1, GP)

In practice J, GPs discussed how they had changed their approach to delayed prescriptions because of the study:

*‘We dispense to ninety-nine point five per cent of our patients* [but] *we came up with a plan. We give the Treating Your Infection leaflet and mark on there when and where to come and get the antibiotic and then the patient could come straight to the dispensary. ’*

(J2, GP)

Interviewees from practice J also mentioned how they spoke about delayed prescriptions differently because of the study materials:

*‘*[Previously] *I would have said, if it doesn’t get better in forty-eight hours come back. But I found it very helpful to say we don’t know what a natural course of a disease is and if things change, then it may be appropriate to use. ’*

(J1, GP)

### POC-CRPT

All practices were interested in using POC-CRPTs. Eight practices accepted Afinion equipment, which recorded all tests run (Table 4).

**Table 4.** The number of Afinion POC-CRP tests carried out per practice and results, as recorded on Afinion machines

**Practice**	**Tests run, *n***	**Lowest result**	**Highest result**	**Results by CRP range,*n* (%)**			
				**≤20^a^**	**21–50**	**51–99**	**≥100**
**A**	54	<5	101	47	5	1	1
**B**	2	<5	20	2	0	0	0
**C**	3	<5	36	2	1	0	0
**D**	4	19	67	1	3	0	0
**E**	70	<5	85	51	13	6	0
**F**	81	<5	163	55	17	5	4
**G**	82	<5	>200	61	12	6	3
**H**	13	<5	60	8	3	2	0
**J**	N/A	N/A	N/A	N/A	N/A	N/A	N/A
**Total**	309	N/A	N/A	227 (73)	54 (17)	20 (0.7)	8 (0.3)

a *National Institute for Health and Care Excellence guidance states that for CRP results of less than 20 the patient is unlikely to benefit from antibiotics.^21^ CRP results of 100 and over are likely to benefit from antibiotics. For CRP results between 20–100, a prescriber should consider a delayed antibiotic prescription. CRP = C-reactive protein. POC-CRP = point-of-care CRP. N/A = not applicable.*

Four practices (A, E, F, G) ran >50 Afinion POC-CRPTs. Most tests gave CRP values ≤20 (73%). No tests were run after March 2020. Practice J were provided with 60 SureScreen tests and had 20 remaining.

At the 2-month survey, most prescribers reported having used POC-CRPTs (26/35, 74%); most had used Afinion only (18/26, 69%). Most prescribers (22/35, 63%) agreed POC-CRPTs had helped reduce antibiotic prescribing.

Interviewees in four practices (A, E, F, G) reported that the Afinion machine was used by one person. Some practices had one GP referring patients to a nurse or ambulatory clinician to have a POC-CRPT and one had one GP doing tests on his own patients:

*‘No one’s been trained to do it, none of the GPs really know how to do it, so I’m the only one that’s trained, so if I’m not here, they’re not able to do it, and if I’m here, then that’s when I’ll do the testing for them. ’*

(A1, non-prescriber)

In all four practices only one GP’s prescribing was being influenced by the POC-CRPT; other prescribers did not participate in using, or referring patients to, the test. Practice B had also allocated the Afinion machine to a nurse but only recorded two tests being conducted. Other practices had POC-CRPT equipment available to all staff but reported infrequent use. In practice D, the Afinion machine was not used at all (except as part of training) because they could not find a suitable place to keep it.

Interviewees reported that they carried out POC-CRPTs mostly on patients presenting with cough, but some practices also included patients with other conditions, indicating mission creep. (Interview participants mentioned using POC-CRPTs for: polymyalgia rheumatica, chronic obstructive pulmonary disease, abdominal pain to check it was not diverticulitis, to rule out acute pancreatitis, and knee pain to rule out septic arthritis.) Participants most often discussed using POC-CRPTs to convince patients they did not need an antibiotic, although some did use it when uncertain:

*‘Our patients will demand antibiotics and so we found the testing extremely, extremely useful for that because once you could give them the result and say, look, antibiotics really won’t be useful, they seem to accept that more than the explanation. I think we’re pretty confident that our prescribing did go down. ’*

(E1, GP)

Two practices (C, F) mentioned that the SureScreen lateral flow POC-CRPT was more practical than Afinion during home visits or when they wanted to avoid leaving their consultation room.

In summary, results indicated that practice J had engaged with implementation as intended and had chosen to use all three AMS strategies in a way that worked for their practice (Box 1), practices A, E, F, and G had engaged partially with implementation (focusing on the physical resources of POC-CRPTs and leaflets), and the remaining practices had engaged very little.

**Box 1.** A summary of engagement reported by participants from practice J

**Engagement** The antibiotic champion was a GP partner with time allocated to administrative tasks that could be spent on study activities. They were reported to be enthusiastic and effective at getting colleagues engaged with the study. They encouraged a team approach and set the precedent that everyone was expected to contribute. The champion looked at the antibiotic optimisation website closely and used the presentation slides provided to run the practice meeting. The practice meeting covered all three antimicrobial stewardship strategies and attendees discussed how each would work in their practice. Practice J was the only practice not to use the Affinion point-of-care C-reactive protein test (POC-CRPT) as they deemed it impractical to use one machine. The practice team made decisions to put SureScreen POC-CRPTs and leaflets in each consultation room so clinicians would have them to hand. The group also decided on how they would consistently issue delayed prescriptions. In interviews, both the champion and another member of staff displayed detailed knowledge of the antibiotic optimisation website. Practice J was the only practice where interview participants acknowledged the specific communication strategies (as detailed in the website) to discuss no antibiotic decisions and delayed prescriptions.

## DISCUSSION

### Summary

Prescribing data indicated no evidence of change overall, or by antibiotic type. Practice J did not appear to reduce their prescribing, although their prescribing remained steady as opposed to other practices where prescribing rose. Engagement with intervention materials differed substantially between champions, with website engagement being poor. Lack of time and competing priorities in general practice were frequently cited as reasons for low engagement.

Champions in five practices initiated changes to adopt AMS strategies, most often the POC-CRPT, which was used most frequently when allocated to one person.

### Strengths and limitations

The study emulated a real-life scenario with an intervention delivered remotely with minimal interaction between practices and researchers. Complementary AMS strategies were available to prescribers with choice to use all or some. This allowed us to assess how interventions were received outside of a trial setting. High-prescribing practices were selected to represent practices who had likely not previously engaged with AMS initiatives. Use of intervention materials could have been monitored more closely by visiting practices, although this may have influenced behaviour.

### Comparison with existing literature

The discrepancy between participant reports of improved prescribing and actual prescribing rates may be explained by the use of routine data and inability, within these data, to identify antibiotic use for specific indications. Previous research has shown positive effects of AMS strategies on antibiotic prescribing for RTIs, but not on antibiotic prescribing overall; this may apply to this study.^22^ Researchers have called for better diagnostic coding.^23^ Data also indicated that communication across teams was poor, with intervention materials often only supporting the prescribing decisions of one clinician.

Five practices had implemented at least one AMS strategy, indicating that initial adoption of strategies in high-prescribing practices is possible, although further optimisation is clearly required to improve prescribing. Previous research has emphasised the importance of champions;^22^^,^^24^ however, implementation was often conducted as a one-off brief activity where decisions caused minimal disruption to existing ways of working, mainly providing additional resources to be used as desired. In some practices champions had adopted strategies themselves, but not informed others, as seen elsewhere.^25^

Champions appeared to see their role as time limited and predominantly focused on raising awareness, rather than encouraging active engagement over time, contrasting with previous work on champions as catalysts for dissemination of innovation.^26^

The physical resources were used most by participants, highlighting the importance of environmental cues. These not only acted as reminders of AMS but also provided new tools, readily available at the time of the prescribing decision, to allow clinicians to approach consultations differently (behavioural substitution).^27^

Access to POC-CRPTs had been a particular motivation to join the study and practices were primed to receive this strategy. However, despite evidence that POC-CRPTs were used, how they were used appeared suboptimal. The antibiotic optimisation website specified that POC-CRPTs were most valuable when there was diagnostic uncertainty or when considering prescribing (scenarios used in trials).^6^^,^^7^

It was encouraging to see that most CRP results were low (<20). However, like previous research, participants reported that POC-CRPT was most often used to convince patients of a no antibiotic decision, so low test results were perhaps unsurprising.^28^^,^^29^ Although this may have reduced prescriptions by avoiding prescribers ‘giving in’ to patients, POC-CRP testing was not used to its full potential and such use is arguably an expensive form of communication, particularly if it lengthens consultations.^7^^,^^30^

It is also important to recognise that clinicians may overestimate patient expectations for antibiotics and, as such, use of a POC-CRPT to ‘convince’ patients that an antibiotic is not required may be misplaced at times.^30^^–^^32^ Participants also reported using POC-CRPTs for a range of presentations, indicating mission creep, again seen in previous work.^28^

Lack of engagement with the website meant prescribers did not know how AMS strategies could benefit them and their patients. The communication training (‘Finding the right words’) had been named to appeal to prescribers, who report difficulties in discussing prescribing decisions.^11^^,^^30^^,^^33^

Despite being approved by clinicians during intervention development, this content was misperceived as something that prescribers already do; however, from observational work it is known this is not done consistently.^34^

Delayed prescriptions were viewed in the same vein. As a result, prescribers did not see a discrepancy between their current behaviour and the desired behaviour.

### Implications for practice

Champions are needed until new ways of working become ingrained. Champions are often self-selecting and internally motivated to undertake additional activities. This role is hard to replicate in high-prescribing practices where there are competing priorities without additional resource.^23^^,^^35^ There may therefore be benefit to having champions outside the practice team, and, if so, the authors of this study would advocate that they should have easy and regular access to prescribers to be able to review and give feedback on adoption and use. The champion’s role should be defined as a longer-term position and appropriately supported.

Champions in the current study did not engage all prescribers in their practices, which is likely to be a continuous challenge with increases in part-time working and staff turnover. Rather than training all prescribers to be fluent in all AMS strategies, it may be more feasible to triage patients with acute infections to specific individuals or teams who are trained and supported to use a breadth of AMS strategies. Such teams may utilise nurses and allied healthcare professionals, and incorporate continued professional development activities.

Interventions that are delivered remotely and passively meet challenges in how they are received in primary care. Physical resources delivered in the current study were given most attention as additions to the environment. In contrast, the website was overlooked. Online interventions are likely to be better received if incorporated into continued professional development programmes and existing electronic systems; however, this needs to be in line with existing workflows to avoid adding burden. In-person training is likely required, either with champions or practice teams, to ensure introductory messages about AMS strategies are received. An example is the TARGET ‘Train the Trainers’ scheme.^36^ Such training allows opportunity to address preconceptions about strategies and specify how they can be used for greatest benefit.

Introduction of POC-CRPTs runs the risk of it being used in practice to support communication rather than reduce diagnostic uncertainty. A general practice consultation may therefore not be the best environment for such diagnostics. NHS England is encouraging POC testing in community pharmacies; and tests to support management of acute infections may be a useful addition here, especially if POC tests are included in service contracts to guide use.^37^^–^^39^

POC testing in locality hubs, which specialise in the management of acute infections, is another possibility. Longer term, there may be potential to have POC testing in the home to support patient self-management.

In conclusion, there was no evidence that providing an intervention to support practices where there is high antibiotic prescribing to adopt AMS strategies affected antibiotic prescribing.

Although some strategies were adopted over others this was not an informed choice because of lack of engagement with web-based resources. POC-CRP testing was most often used as a novel tool. When AMS strategies were used, their use was often suboptimal (compared with use in trials), missing out on additional benefits. AMS strategies are complex interventions and require clinicians to have detailed knowledge on how to adopt them in practice if they are to achieve benefits in reducing antibiotic use.

Successful adoption may be achieved by triaging patients and allocating one or two people to use AMS strategies. Remote, one-sided provision of AMS strategies should be used cautiously with engagement monitored to ensure optimal reception.

## Data

The datasets analysed during this study are available from the corresponding author on reasonable request.
